# Long-term follow-up of a randomized controlled trial of Lichtenstein repair vs the Valenti technique for inguinal hernia

**DOI:** 10.1007/s10029-019-01879-y

**Published:** 2019-01-18

**Authors:** K. Mitura, K. Garnysz, I. Michałek

**Affiliations:** 1 General Surgery Department, Siedlce Hospital, ul. Narutowicza 25, 08-110 Siedlce, Poland; 2 Department of Health Sciences, University of Natural Sciences and Humanities, Siedlce, Poland

**Keywords:** Inguinal hernia, Mesh, Recurrence, Hernia repair, Follow-up

## Abstract

**Purpose:**

The aim of the study was to offer a prospective comparative assessment of long-term outcomes for inguinal hernia repair using Valenti and Lichtenstein techniques.

**Materials and methods:**

568 surgical procedures for unilateral inguinal hernia repair using the Valenti (group V) or the Lichtenstein technique (group L) were performed. After the mean follow-up time of 9 years (8–12), 185 patients (70.1%) treated using Valenti method and 186 patients (71.3%) treated using Lichtenstein method were clinically assessed. All clinical data were registered in National Hernia Registry. The rate of recurrence was assessed as primary outcome. The secondary outcome involved chronic pain (VAS).

**Results:**

9-year recurrence rate was 2.2% in both groups. No significant difference in recurrence rate was demonstrated in analysis adjusted for surgeon’s education, type of hernia, hernia size, hernia duration, or BMI between two groups (OR 1.0; 95% CI 0.69–1.67; *p* = 1.0). In follow-up the majority of patients reported no pain (71.9% in V; 73.7% in L). A constant pain was reported by four patients in each group. Severe pain was reported by 1.6% in V and 2.1% in L (*p* = 0.192).

**Conclusions:**

Inguinal hernia repairs using Valenti and Lichtenstein methods show high, long-term effectiveness and do not significantly differ in the recurrence rate. Both methods ensure a low rate of chronic pain. The use of a single mesh size with a precisely defined shape and of a uniform mesh fixation method ensures the standardization of surgical technique. The Valenti method is an uncomplicated, technically reproducible procedure with a low learning curve.

## Introduction

Inguinal hernia repair is one of the most common surgical procedures. It is estimated that inguinal hernia occurs at some point during the lifetime of one in four men [1]. For that reason, solving the problem of recurrence associated with pure tissue repairs is very important. The introduction of an artificial material—a polypropylene mesh—was a milestone in hernia repair. Of the many open surgery techniques applied, the Lichtenstein repair using a synthetic implant to strengthen the posterior wall of the inguinal canal is currently recognized as the “gold standard” [2]. The Lichtenstein technique is based on the principle of having no tension in the line of sutures.

Based on observations made in cases of repair procedures performed due to recurrences after these surgeries, the postoperative deformation of the implanted mesh is often notable [3]. The deformation is caused by, among other things, changed position of the patient’s body directly after the surgery and tension in the muscles to which the mesh is fixed. These tensions lead to creasing and wrinkling of the synthetic material and the fixation of that shape by fibrotic tissues. Additionally, despite the common use of the Lichtenstein technique, there are numerous possible modifications of the method. This applies mainly to the size of the mesh, the shape, type, and location of sutures, the method of making the incision or opening for the spermatic cord, the way the mesh tails are positioned and fixed, etc. It has been shown that despite the majority of surgeons declaring that they use the original Lichtenstein method, they use techniques that differ greatly in detail.

The proposal of a solution unified in technical details that takes into account changes in the topography of the groin area as a result of verticalization of the patient after the surgery and of muscle tension was presented in 1999 by the Italian surgeon Gabrielle Valenti [4]. He used a flat polypropylene mesh with a precisely defined shape and dimensions and consisting of two complementary flat elements. Only one edge of each mesh was fixed to surrounding tissues during the surgical procedure. With this technique, during postoperative patient movement, the mesh is held on one side only, which leaves it flat and non-deformed. After obtaining the appropriate tension characteristics for a particular patient, the mesh automatically adapts to the individual anatomical conditions of the groin area. In this formation, the mesh is overgrown by connective tissue, which fixes its flat shape with no additional tension.

A lack of tension in the suture line and the maintenance of the correct flat shape of the prosthesis following the repair procedure, according to the author’s method, reduces pain during the postoperative period and reduces the number of recurrences [5].

## Purpose

The aim of the study was to offer a prospective comparative assessment of long-term outcomes for inguinal hernia repair using the Valenti and Lichtenstein techniques.

## Materials and methods

### Patients

**Fig. 1:**
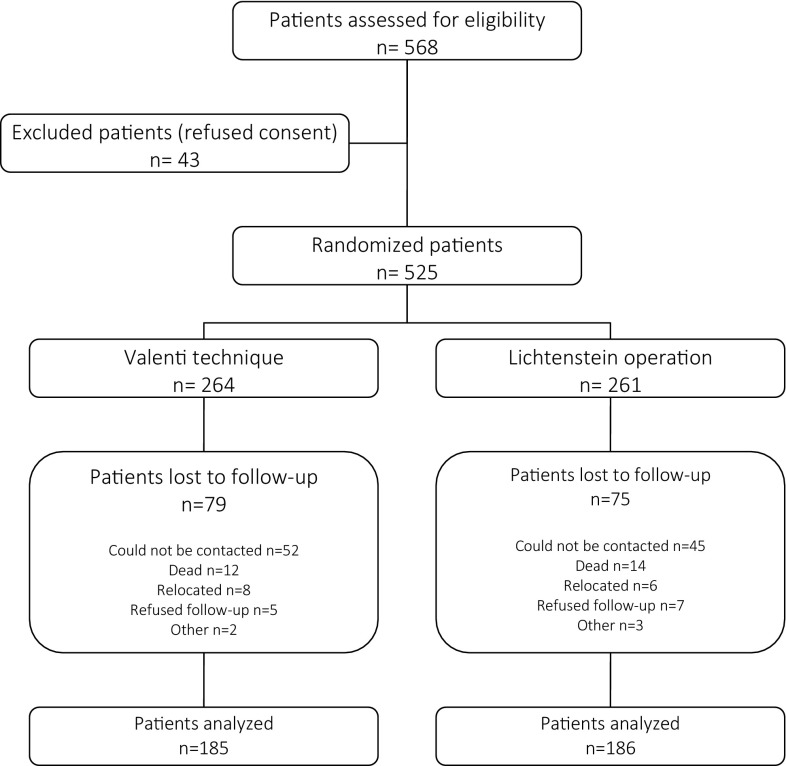
Consort diagram

From September 2006 to June 2010, patients with primary or recurrent inguinal hernias were simply randomized with number tables to undergo Valenti or Lichtenstein surgery. The study was approved by the local ethics committee. A total of 568 surgical procedures for unilateral inguinal hernia repair using the Valenti technique (group V) or the Lichtenstein technique (group L) were performed during the study period. Patients with bilateral inguinal hernias also qualified for the study, but only one side was surgically repaired. Patients with incarcerated hernias or recurrent hernias after mesh repair and patients with mental disorders were excluded from the study. Before the surgery, patients were informed in detail about both surgical methods and randomization principles. Forty-three patients refused participation in the randomization. A total of 525 patients were included and randomized (Fig. 1). The study group characteristics are presented in Tables 1 and 2.

**Table 1:** Demographic details of patients with and without 9-year follow-up

	Patients with 9-year Follow-up		Patients Lost to Follow-up	
	Valenti	Lichtenstein	Valenti	Lichtenstein
Patients, *n* (%)	185 (49.9%)	186 (50.1%)	79 (52.3%)	75 (47.7%)
Age, mean (SD), years	50.9 (15.8)	51.2 (15.6)	54.5 (16.9)	54.9 (17.1)
Gender, *n* (%)				
Male	240 (90.9%)	236 (90.4%)	73 (92.4%)	70 (93.3%)
Female	24 (9.1%)	25 (9.6%)	6 (7.6%)	5 (6.7%)
BMI, mean (SD), kg/m^2^	26.2 (2.9)	26.4 (2.8)	25.9 (3.0)	26.2 (2.7)
ASA				
I	106 (57.3%)	110 (59.2%)	46 (58.2%)	45 (60.0%)
II	68 (36.8%)	65 (34.9%)	26 (32.9%)	25 (33.3%)
III	11 (5.9%)	11 (5.9%)	7 (8.9%)	5 (6.7%)
Smoking, *n* (%)				
Yes	109 (58.9%)	112 (60.2%)	45 (57.0%)	42 (56.0%)
No	76 (41.1%)	74 (39.8%)	34 (43.0%)	33 (44.0%)
Job type, *n* (%)				
Manual	72 (38.9%)	69 (37.1%)	28 (35.4%)	25 (33.3%)
Sedentary	39 (21.1%)	43 (23.1%)	19 (24.1%)	17 (22.7%)
Retired/unemployed	74 (40.0%)	74 (39.8%)	32 (40.5%)	33 (44.0%)
Hernia duration, *n* (%)				
< 12 months	115 (62.2%)	113 (60.8%)	47 (59.5%)	44 (58.7%)
1–5 years	50 (27.0%)	47 (25.3%)	21 (26.6%)	19 (25.3%)
> 5 years	20 (10.8%)	26 (13.9%)	11 (13.9%)	12 (16.0%)
Hernia reducibility, *n* (%)				
Yes	172 (93.0%)	171 (91.9%)	70 (88.6%)	68 (90.7%)
No	13 (7.0%)	15 (8.1%)	9 (11.4%)	7 (9.3%)
Recurrent hernia, *n* (%)				
Yes	17 (9.2%)	11 (5.9%)	4 (5.1%)	4 (5.3%)
No	168 (90.8%)	175 (94.1%)	75 (94.9%)	71 (94.7%)
Preoperative hernia size				
Above inguinal ligament	95 (51.3%)	102 (54.8%)	43 (54.5%)	43 (57.3%)
Below inguinal ligament (excluded scrotal)	64 (34.7%)	61 (32.9%)	28 (35.4%)	22 (29.3%)
Scrotal < 5 cm	11 (5.9%)	10 (5.4%)	3 (3.8%)	3 (4.0%)
Scrotal 5–10 cm	13 (7.0%)	9 (4.8%)	3 (3.8%)	5 (6.7%)
Scrotal > 10 cm	2 (1.1%)	4 (2.1%)	2 (2.5%)	2 (2.7%)

**Table 2:** Basic characteristics of procedures in patients with and without 9-year follow-up

	Patients with 9-year Follow-up		Patients Lost to Follow-up	
	Valenti	Lichtenstein	Valenti	Lichtenstein
Procedures, *n* (%)	185 (49.9%)	186 (50.1%)	79 (52.3%)	75 (47.7%)
Anesthesia, *n* (%)				
Local	10 (5.4%)	9 (4.8%)	3 (3.8%)	5 (6.7%)
Spinal	157 (84.9%)	161 (86.6%)	63 (79.7%)	61 (81.3%)
General	18 (9.7%)	16 (8.6%)	13 (16.5%)	9 (12.0%)
Hernia type, *n*				
Direct (M1/M2/M3)	65 (24/13/28)	70 (27/13/30)	33 (12/6/15)	31 (11/7/13)
Indirect (L1/L2/L3)	134 (47/39/48)	132 (44/43/45)	63 (20/19/24)	60 (22/16/22)
Surgeon, *n* (%)				
Resident	44 (23.8%)	47 (25.3%)	20 (27.8%)	18 (24.0%)
Attending surgeon	141 (76.2%)	139 (74.7%)	59 (72.2%)	57 (76.0%)
Operation time, mean (SD), min	52.3 (17.2)	54.5 (15.3)	54.6 (16.8)	51.2 (15.8)
Nerve resection, *n* (%)				
Iliohypogastric	44 (23.8%)	42 (22.6%)	19 (24.0%)	17 (22.7%)
Ilioinguinal	4 (2.2%)	6 (3.2%)	2 (2.5%)	2 (2.7%)
Genital branch of femoral	0 (0.0%)	0 (0.0%)	0 (0.0%)	0 (0.0%)

### Treatment

The standard protocol for surgical preparation, which involved prophylactic antibiotic administration (1.0 g cefazolin I.V. at the time of surgery for patients < 80 kg, and 2.0 g for patients over 80 kg), was used. The type of anesthesia was chosen by an anesthesiologist, considering the patient’s preference. Surgical procedures were performed under regional anesthesia (84.9 vs 86.6% in groups V and L, respectively), general anesthesia (9.7 vs 8.6%), or local anesthesia (5.4 vs 4.8%). An experienced senior surgeon participated in each procedure as an operating or assisting surgeon. Randomization was performed using a computer-generated selection provided in a non-marked, sealed envelope opened at the time of the surgery.

Surgical procedures were performed according to the original techniques proposed by the authors of each method [4, 6]. A 10 × 15-cm polypropylene mesh (Ultrapro; Ethicon, Somerville, NJ, USA) was used in the Lichtenstein surgery. The mesh was trimmed to a shape fitting the bottom of the inguinal canal. The inferior edge of the mesh was fixed to the inguinal ligament from the pubic tubercle to the level of the deep inguinal ring using a continuous 2/0 monofilament non-absorbable suture (Surgipro; Covidien, Mansfield, MA, USA). The superior-medial edge was fixed with two or three single sutures.

**Fig. 2:**
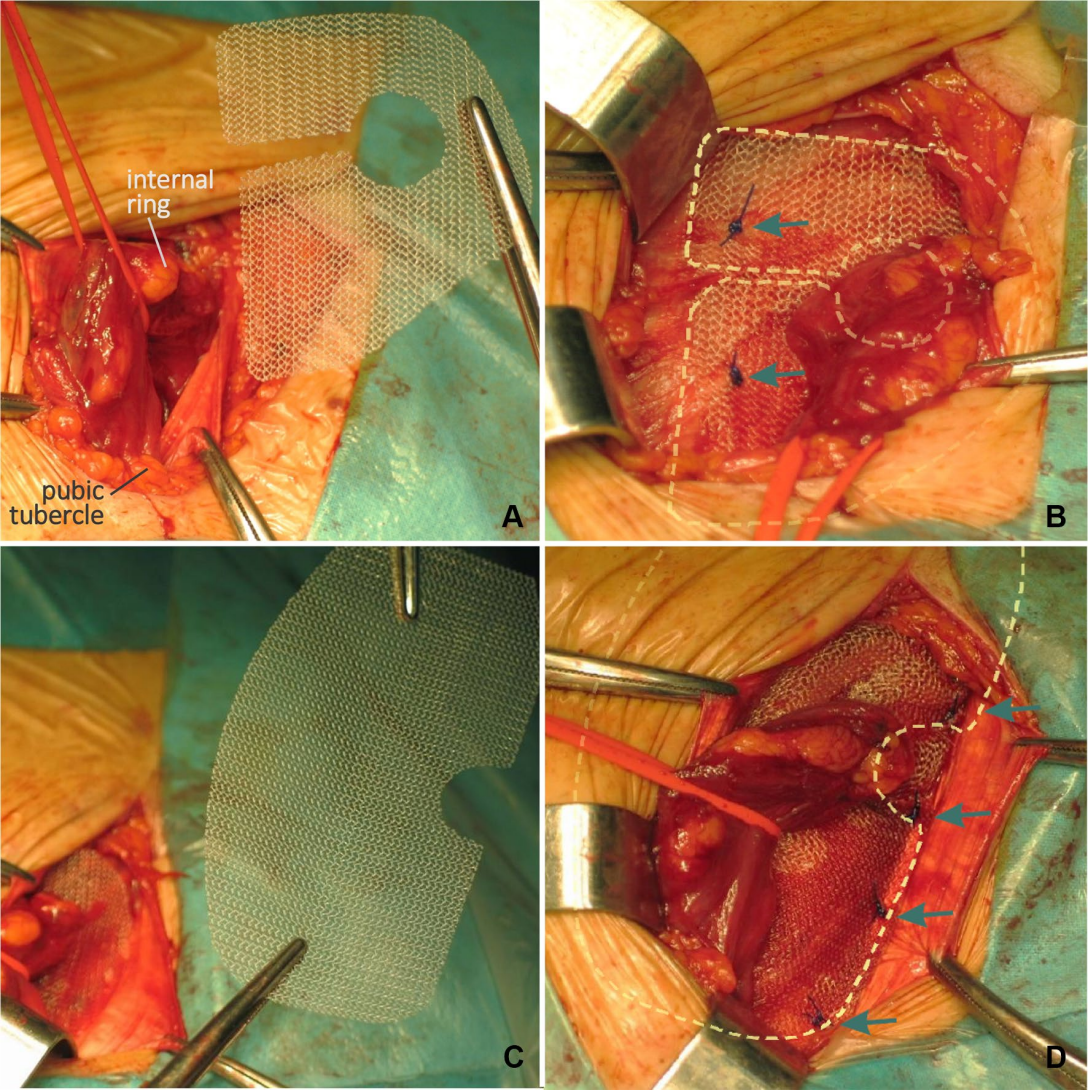
Valenti hernia repair of a left inguinal hernia. Arrows indicate fixating sutures. Trapezoid mesh (**a**) located around the internal ring (**b**) on the posterior wall. Rectangular mesh (**c**) implanted directly over the previous mesh. The spermatic cord passes through a semilunar incision (**d**). Dotted lines indicate the shape of the meshes

The Valenti technique used a pre-shaped polypropylene mesh (Aspide Medical; La Talaudiére, France). The set is composed of two complementary meshes—a smaller trapezoid-shaped mesh (4.5 × 5 cm) with an opening for the spermatic cord and a larger rectangular mesh (6 × 11 cm) with rounded corners and a semi-circular cut for the spermatic cord on its inferior edge (Fig. 2). The first, trapezoidal fragment of the mesh is placed on the posterior wall of the inguinal canal so that the opening in the mesh surrounds the spermatic cord at the level of the deep inguinal ring. Two branches of that component should be perpendicular to the midline, and both tails should be fixed to the anterior rectus sheath with a single, non-absorbable, 2/0 monofilament suture (Surgipro). The other, rectangular component of the mesh is placed over the first so that its inferior edge with a semi-circular cut for the spermatic cord was parallel to the inguinal ligament. Four single, non-absorbable sutures are placed on that edge of the implant only. The first suture fixes the corner of the mesh to the pubic tubercle. Another two sutures fixing the mesh to the inguinal ligament are placed on the edges of the semi-circular cut, with the spermatic cord passing through it. The last suture is placed midway between the two sutures placed medially from the semi-circular cut. Then, the aponeurosis of the abdominal external oblique muscle is sutured over extended components of the mesh, thus re-creating the anterior wall of the inguinal canal and the superficial inguinal ring.

### Follow-up

The course of the surgical procedure and of the postsurgical period was analysed. All patients were assessed daily for two days after the repair surgery and one week after in the outpatient setting. After the mean follow-up time of 9 years (8–12), 185 patients (70.1%) treated using the Valenti method and 186 patients (71.3%) treated using the Lichtenstein method were clinically assessed. In most cases, the reason for the loss to follow-up was a lack of contact, a non-treatment-related death, relocation, or refusal to give an answer (Fig. 1). A comparison of groups of patients lost to follow-up and of fully clinically assessed patients is presented in Table 1.

All clinical data were registered in the National Hernia Registry. The rate of recurrence was assessed as the primary outcome in the study groups. The secondary outcome involved the presence of chronic pain in the VAS and a descriptive assessment.

### Statistical analysis

Student’s *t* test and the Mann–Whitney *U* test were used for the analysis of qualitative data. The normality of distribution was verified using the KS test. Pearson’s v2 and Fisher’s exact tests were used for the analysis of qualitative data. Differences were considered statistically significant if *p* < 0.05. Calculations were performed using SPSS version 25.0 software (SPSS, Chicago, IL, USA).

## Results

### Primary outcome

The 9-year recurrence rate was 2.2% (4/185 hernias) in group V and 2.2% (4/186) in group L. No significant difference in the recurrence rate was demonstrated in the analysis adjusted for the surgeon’s education, type of hernia (primary vs recurrent), pre-surgical assessment of the hernia size, hernia duration, or BMI between the two groups [OR 1.0; 95% CI 0.69–1.67; *p* = 1.0 (Table 3)]. Among all eight cases of recurrence, two occurred in patients with recurrent hernia—one in each group. Only five patients were subject to another surgical procedure because of a recurrence (two cases in group V and three in group L) throughout the 9-year follow-up period.

**Table 3:** Multivariate analysis of recurrence rate at 9-year follow-up

Covariate	Univariate analysis		Multivariate analysis	
	OR (95% CI)	*p*	OR (95% CI)	*p*
Valenti vs Lichtenstein	1.01 (0.68–1.65)	0.979	1.00 (0.69–1.67)	1.00
Resident vs attending surgeon	1.04 (0.59–1.97)	0.716	1.05 (0.61–1.99)	0.748
Recurrent vs primary hernia	1.57 (0.68–2.39)	0.391	1.52 (0.66–2.25)	0.402
Scrotal vs non-scrotal hernia	1.15 (0.65–1.98)	0.294	1.09 (0.62–1.90)	0.301
Hernia duration < 1 year vs > 1 yr	0.98 (0.52–1.67)	0.288	0.99 (0.53–1.71)	0.297
BMI (per 1 unit)	1.12 (0.88–1.38)	0.241	1.11 (0.89–1.40)	0.249

### Secondary outcomes

In the long-term follow-up in both study groups, the majority of patients reported no pain (71.9% in group V and 73.7% in group L) (Table 4). For patients reporting the presence of pain, a constant pain was reported by four patients in each group. The ratio of people complaining of severe pain was 1.6% in the V group and 2.1% in the L group (*p* = 0.192). We could not find a difference in chronic pain between the groups in an analysis adjusted for nerve management, type of anesthesia, surgeon’s experience or hernia size (Table 5). Seroma occurred in 12 patients (6.6%) in group V and in 14 patients (7.6%) in group L, and the difference was not statistically significant (*p* = 0.285). Drainage was not necessary in any case, and fluid collections were absorbed spontaneously.

**Table 4:** Sensory disorders and pain in the analysed groups after 9 years of follow-up

	Valenti	Lichtenstein	*p*
	*n* = 185	*n* = 186	
VAS, mean (SD)	0.43 (0.41)	0.39 (0.38)	0.485
Verbal description of pain, *n* (%)			
No pain	133 (71.9%)	137 (73.7%)	0.283
Mild pain	41 (22.2%)	36 (19.4%)	0.524
Moderate pain	8 (4.3%)	9 (4.8%)	0.331
Severe pain	3 (1.6%)	4 (2.1%)	0.192
Pain occurrence, *n* (%)			
No pain	133 (71.9%)	137 (73.7%)	0.229
Incidental	48 (25.9%)	45 (24.2%)	0.362
Constant pain	4 (2.2%)	4 (2.1%)	0.664
Foreign body sensation, *n* (%)	25 (13.5%)	23 (12.4%)	0.536
Loss or change of sensation, *n* (%)	51 (27.6%)	56 (30.1%)	0.098

**Table 5:** Multivariate analysis of recurrence rate at 9-year follow-up

Covariate	Univariate analysis		Multivariate analysis	
	OR (95% CI)	*p*	OR (95% CI)	*p*
Valenti vs Lichtenstein	0.88 (0.39–1.97)	0.116	0.89 (0.41–1.99)	0.119
Nerve resection vs no resection	0.63 (0.33–2.01)	0.074	0.64 (0.34–1.97)	0.075
Local anesthesia vs spinal/general	0.92 (0.51–1.83)	0.163	0.91 (0.53–1.90)	0.166
Resident vs attending surgeon	1.08 (0.63–2.45)	0.259	1.08 (0.61–2.48)	0.261
Scrotal vs non-scrotal hernia	1.12 (0.73–1.95)	0.337	1.10 (0.69–1.96)	0.333

## Discussion

The concept of the PAD method (from Italian: Protesi Autoregolantesi Dinamica) introduced by the Italian surgeon Gabrielle Valenti in 1992 is based on an attempt to combine advantages of the Lichtenstein technique (low recurrence rate) with the comfort achieved following repair surgeries involving no suturing (minimal pain) [6]. In 1999, Valenti presented the results of treatment of a group of 500 patients with the new method [4]. At that time, he paid attention primarily to minor pain in the postoperative course. For the majority of patients, painkillers were necessary only on the first day after the repair procedure. Similar results were obtained by us. From the second postoperative day onwards, patients scored their pain below 3 on the VAS scale [7]. Based on an analysis of 585 hernioplasties, Valenti reported the presence of pain in just 5.1% of patients after 4 weeks and of persistent neuralgia in just 0.3% [5]. A small percentage of patients with persistent pain may be the result of using only two single sutures securing the mesh to the rectus sheath in the Valenti method. This avoids damage to the iliohypogastricus and the ilioinguinalis nerve fibers, which is possible in the Lichtenstein method [8, 9]. Other applied sutureless methods are also associated with less pain in the early postsurgical period. The comfort associated with the use of sutureless methods (e.g., PHS, UHS) is a result of the absence of nerve fiber compression. However, in those methods, a component of the mesh is introduced into the preperitoneal space. Treatment in this space is extremely difficult in cases of mesh infection [10]. The Valenti method does not involve interference with the preperitoneal space, and thus, these complications may be avoided. Additionally, newly introduced techniques involving adhesives for mesh fixing or self-gripping meshes offer the potential to minimize postsurgical pain, but there are concerns regarding their effect on the recurrence rate [11, 12].

In the presented material, Valenti did not find any recurrent hernia [4]. This may be due to the lack of tissue tension within the surgical area, which is consistent with the current guidelines for the treatment of hernias. A change in body position is associated with a significant change in the relative position of the inguinal ligament and broad muscles of the abdomen. The distance between the inguinal ligament and the rectus muscle is increased, and the abdominal wall bulges. For this reason, securing the mesh components to only one edge makes the mesh rest freely on the posterior wall of the inguinal canal, and after the patient changes position, it assumes a natural, free position, and only in this optimal position is the mesh overgrown by connective tissue. This avoids uncontrolled creasing of the mesh, which is possible with the Lichtenstein method. Maintaining the flat shape of the mesh components results in protection of the entire surface of Hesselbach’s triangle, thus preventing relapse.

Performing inguinal hernia repair using the Valenti method is an uncomplicated procedure. Valenti uses prefabricated polypropylene mesh components with a precisely defined shape. The use of a single standard model eliminates the need to trim mesh as in the Lichtenstein surgery. The size and shape of the meshes in the Valenti method were developed on the basis of anthropometric studies and constitute a universal solution for adult patients [4]. The use of only six single sutures securing the mesh components to anatomical structures in precisely defined places eliminates the variability associated with methods of mesh securing applied in other methods (continuous or single sutures, more or less dense points of passing the needle, etc.). Principles defined in the Valenti method result in uniformization of the course of the surgical procedure, even if performed in different centers. Simple principles and the absence of technical challenges make this surgical procedure clear, logical and easy to complete, even for less-experienced surgeons. The most important advantage of the Valenti method is the maintenance of all general assumptions of the Lichtenstein method [4, 6]. Those assumptions are a 2-cm margin over the pubic tubercle, at least a 3-cm margin beyond Hasselbach’s triangle, and a 5-cm mesh margin lateral to the deep ring. Therefore, the entire area of reduced strength is fully covered with mesh.

The use of two meshes in the Valenti method results in the creation of a valve system immediately after the surgery, reinforcing the area of the internal ring. A similar mechanism is used in the Desarda method, offering outcomes similar to those of the Lichtenstein method, despite the absence of a mesh [13]. This is also the reason for not creating a spermatic cord opening in the Lichtenstein method. In the original method, tails of the mesh are crossed, creating a valve mechanism suspending the spermatic cord at the level of the deep inguinal ring [4, 6]. In the Valenti method, with the increasing tension of abdominal muscles, the smaller mesh pulls the internal ring medially and upwards, while the larger mesh with the semi-circular cut holds the spermatic cord downwards and laterally (towards the inguinal ligament). Thus, both components form a valve preventing the bulging of the peritoneum.

Initially, we were concerned about the necessity for using two meshes, one on top of the other. This raises concerns about the increased risk of infections or the development of a seroma [14]. However, the results presented by Valenti indicated an absence of that type of correlation. It seems that a total absence of tension and creasing is responsible for that mechanism. In other methods involving the use of meshes, surgeons usually try to avoid creasing or stacking meshes due to concerns about dead spaces between mesh fibers. The results of our previous observations confirmed that laying two meshes flat, with no tension, one over the other, does not increase the risk of seroma and infections, even without the use of macropore meshes [7].

Recurrences after inguinal hernia repair may occur even after 5 years of follow-up, at a rate close to 4% [15]. However, that is still a significantly lower rate compared to that of suture repair. Hence, the number of recurrences is the basic parameter used for the assessment of the treatment outcome. Despite using a smaller mesh size than that usually used in the Lichtenstein method (6 × 11 cm for the Valenti vs 10 × 15 cm for the Lichtenstein method), the Valenti method ensures a low rate of recurrence. We observed four cases of recurrence in each study group, which constituted approx. 2%. The detailed analysis of the circumstances of the recurrence showed that for the Valenti method, in two cases, it was the result of a lack of identification of a concomitant oblique hernia while repairing only the medial defect in the bottom of the inguinal canal (early relapses). A flat position of both meshes and the absence of any deformations was observed during repeated surgical procedures in these patients. Both meshes were fused, and they could no longer be separated. The low, long-term recurrence rate confirms a similar efficacy of both methods.

In the face of similar results for treatment efficacy with both methods, the assessment of long-term pain may be important. It is estimated that pain of various intensities may occur in 0.3–32% of patients. At the same time, strong pain, affecting the patient’s everyday functioning, usually occurs in less than 3% of patients. The precise mechanism responsible for the development of pain is not fully understood, and its multifactorial etiology is postulated [16]. In the analyzed material, we demonstrated that the number of patients suffering persistent pain was similar in both groups. Considering the lack of correlation between chronic pain and the surgical technique, it can be presumed that the use of two complementary meshes in the Valenti method does not result in greater nerve damage that promotes chronic neuralgia.

The limitation of this work is the long-term assessment of just over 70% of patients, although the majority of patients lost to follow-up were not related to the treatment. At the same time, a comparative analysis of groups of patients lost and maintained in the study did not show differences between them. This is the largest available long-term analysis of treatment results using the Valenti method and the first long-term analysis of the use of two flat meshes placed on each other. However, additional studies are needed to confirm the results.

## Conclusions

Inguinal hernia repairs using the Valenti and the Lichtenstein methods show high, long-term effectiveness and do not significantly differ in the recurrence rate. Both methods ensure a low rate of chronic pain.

The use of a single mesh size with a precisely defined shape and of a uniform mesh fixation method ensures the standardization of the surgical technique. The Valenti method is an uncomplicated, technically reproducible procedure with a low learning curve.
